# A Critical View of the King's Fund

**Published:** 1914-10-31

**Authors:** 


*To the Editor of* The Hospital.

### A Critical View of the King's Fund.

A GRUMBLE AND A SUGGESTED DEPARTURE.

*To the Editor of* The Hospital.

Sir,—Appreciation is encouraging, but criticism is sometimes more useful. No one who is interested in hospitals can fail to appreciate the work of King Edward's Hospital Fund for London, or to echo the words of the contributor to The Hospital of October 10 on this subject.

Nothing but good, however, can be served by putting forward some of the criticism which is frequently heard in hospital circles, but does not often find public expression. Statistics are not good things in themselves, but, like dog-whips or dust-bins, merely a means to an end of discipline and good order. The value of the statistical report recently published depends, therefore, upon the reliability of the figures published and the use which can be made of them.

The Uniform System of Accounts has done wonders, but it has not produced a basis which enables a member of the public to judge between the economy and efficiency of different hospitals. He can see that one hospital spends some £90 per occupied bed and another about £60; he may guess that there is some fundamental difference of administration or policy which accounts for such an amazing difference, but he has no guide whatever as to whether he is getting better value for his money in giving to one or the other. If he is influenced by economical considerations, his judgment is swayed by the warning words of the Introduction. If he leans towards efficiency at any price, he may wonder that the cheaper hospitals can escape the criticism of the Fund, or he may reflect that if a bed in the "London" cost only the same as one in a hospital at the bottom of the list, some £30,000 annually would be saved. The subscribing public must depend for guidance, now as in the past, upon the appeals and publications of the hospitals themselves.

No doubt the statistics of the Report are intended mainly for those who are actively engaged in the administration of hospitals. But surely it has become clear to every one of us that the mere figures without an intimate knowledge of the facts underlying them are of little value. In our own case we know that where there is a large variation from the average it is nearly always due to some special reason of which no outsider would be aware, and which frequently makes all comparison with other hospitals impossible. Even assuming that everyone is only concerned with giving an accurate statistical picture of his hospital, the margin of doubt is too great for purposes of comparison. But we all wish not only to give an accurate but also a pleasing picture, and there is undoubtedly plenty of opportunity, without impinging in the smallest degree upon truth or accuracy, of placing doubtful expenditure to the debit of that heading which will show our hospital in the most favourable light.

To take one example, the line between official printing, stationery, etc., and the cost of appeals is often so faint as to be almost indecipherable; while, again, the cost of a special appeal may be charged against the result before the amount is credited to the hospital income, as is regularly done, I believe, in the case of one institution. The same applies to the rent account; where a hospital has large landed property an undue proportion of official salaries may be written off against the rent account, proportionately decreasing the cost of administration as shown in the statistical tables.

Again, where there is a large and flourishing school attached to a hospital, the amount chargeable to either in respect of certain services may be unduly large, or *vice versa*, according to the negotiating power of the parties concerned. These are but a few examples of the general criticism: that the Fund attaches too much importance to statistics and has overloaded the administration with their preparation.

There is too much theory and not enough practical knowledge in their control of hospitals. No practised hospital administrator would ask London hospitals to supply in less than a week the amount of their total expenditure up to a given and very recent date. The thing is impossible; it could not be done. But it has been done because the word of the Fund is law; and the figures supplied must be a matter of guesswork and practically unreliable.

I would like to ask how many hospitals in making this return have included accounts due, but not paid, on the date given, and how many have excluded them. Such inquiries as this are a farce, making a god of the dog-whip and the dust-bin.

But the whole question of how the Fund can best exercise its beneficent control over hospitals is of less importance than the manner in which it can best support and maintain our voluntary system. Its invested funds, its annual grants, are of immense value; while the moral, if not the practical, value of annual inspection can hardly be overstated. There is perhaps a danger that we should under-estimate these benefits because they have become so familiar. But the activities of the Fund surely ought not to stop short at the collection and distribution of this great sum of money. The voluntary system depends not only upon the sympathy but also upon the knowledge of the public; and there is an immense opportunity of educating the public in the work and methods of hospitals. We can see how in a smaller sphere such as Leicester or Newcastle an able committee can arouse local interest and local patriotism until their hospitals rest securely upon knowledge and affection. No single hospital can do the same thing in London, the area is too vast, the Press too general in its character.

But a great central organisation with a well-equipped publicity department could achieve similar results; not necessarily begging, not pleading the cause of any one hospital, but explaining the way in which our system works, its advantages, its difficulties.

If we believe that the Committee of the Fund are convinced that the voluntary system is good and worth maintaining, it is surely well that our reasons for this faith should be known. No one can fail to be struck by the frequency of the pessimistic assumption that the voluntary system is doomed; yet how can we expect to have generous support and eager service from those who believe that they are working for a lost cause? Our cause is not doomed, it is stronger than ever; the Fund itself has two million invested reasons for thinking so—but the public does not know it.

The war has made it clear how few people really understood the work of a nurse, the skill and training it involves, or the methods by which hundreds of women are sent out every year from London hospitals, adequately equipped for the care of every kind of illness or accident.

Surely it is time, too, that certain perennial fictions were finally disposed of, and the staffing of London hospitals explained. I still meet with people who believe that patients in our wards are experimented upon by unqualified students, while the position of house-surgeon is frequently regarded as the highest achievement of a surgeon's ambition. Not one person in a thousand understands the arrangement of the medical and surgical staff or the relation of medical schools to hospitals; and I am speaking of ordinary well-informed people, who might be supposed to have opportunities of knowing, and who are frequently generous subscribers.

Lastly, since we are asking for help from the Committee of the Fund, may we not look to them for more light and guidance in times of crisis and difficulty? The Insurance Act produced, I think, several elaborate forms, but not much guidance, in a new and complicated situation. The war has produced an inquiry form, but we have had so far little attempt to co-ordinate the hospitals and to place the accommodation available at the service of the nation with the least interference with the civil population.

To sum up, then, this attempt at a critical view of an admirable organisation, we need less control in matters of detail, more guidance in matters of principle, less statistical inquiry, more practical inspection—above all things, not less financial help, but more influential assistance in putting the methods and the value of the voluntary system before the public.—I am, etc.,

ASSISTANT HOSPITAL SECRETARY.

October 13, 1914.

